# Arrest and Attack: Microtubule-Targeting Agents and Oncolytic Viruses Employ Complementary Mechanisms to Enhance Anti-Tumor Therapy Efficacy

**DOI:** 10.3390/genes15091193

**Published:** 2024-09-11

**Authors:** Sucheta De, Marcelo Ehrlich

**Affiliations:** Shmunis School of Biomedicine and Cancer Research, George S. Wise Faculty of Life Sciences, Tel Aviv University, Tel Aviv 6997801, Israel; suchetade@mail.tau.ac.il

**Keywords:** oncolytic virus, microtubule-targeting agents, JNK, combination therapy

## Abstract

Oncolytic viruses (OVs) are promising cancer immunotherapy agents that stimulate anti-tumor immunity through the preferential infection and killing of tumor cells. OVs are currently under limited clinical usage, due in part to their restricted efficacy as monotherapies. Current efforts for enhancement of the therapeutic potency of OVs involve their combination with other therapy modalities, aiming at the concomitant exploitation of complementary tumor weaknesses. In this context, microtubule-targeting agents (MTAs) pose as an enticing option, as they perturb microtubule dynamics and function, induce cell-cycle arrest, and cause mitotic cell death. MTAs induce therapeutic benefit through cancer-cell-autonomous and non-cell-autonomous mechanisms and are a main component of the standard of care for different malignancies. However, off-target effects and acquired resistance involving distinct cellular and molecular mechanisms may limit the overall efficacy of MTA-based therapy. When combined, OVs and MTAs may enhance therapeutic efficacy through increases in OV infection and immunogenic cell death and a decreased probability of acquired resistance. In this review, we introduce OVs and MTAs, describe molecular features of their activity in cancer cells, and discuss studies and clinical trials in which the combination has been tested.

## 1. Introduction

Resistance to single-agent therapies remains a major challenge in cancer therapy. Combination therapies allow for the concomitant targeting of multiple hallmarks of cancer [1,2] and diminish the probability of acquired resistance, but they may also compound undesired side effects. The remarkable and diverse arsenal of therapy strategies and agents that have already been developed generates a daunting number of possible combinations. Moreover, to benefit from the untapped potential of such therapy combinations, a thorough understanding of the molecular mechanisms of action of different therapy modalities and the underpinnings of acquired resistance to such therapies is required. Given the predicted scale and complexity of such data and the marked degree of heterogeneity exhibited by different tumors [3,4], the harnessing of the full potential of combination therapies is expected to require artificial intelligence-based analyses. Furthermore, this convolved scenario also generates complexities for the design of clinical trials aimed at probing the efficacy of such combinations [5,6]. In these contexts, the differences in the susceptibility of distinct tumors to different OVs, and the plurality of modes by which OVs target tumors, are expected to further complicate the selection and evaluation of OVs as combination-therapy agents. In the present review, we introduce OVs and MTAs, focusing on the molecular mechanisms by which they exert their effects on tumor cells. Furthermore, we describe and discuss OV-MTA combinations that have been recently tested.

## 2. The Combination Factors: Oncolytic Viruses and Microtubule-Targeting Agents

### 2.1. Oncolytic Viruses and Their Exploitation of the Cancer-Cell Context

Oncolytic viruses are natural or engineered infectious agents capable of selectively replicating in and lysing tumor cells while sparing non-neoplastic tissues [7,8,9,10]. With dependence on OV and/or tumor type, this selectivity may stem from virally encoded elements, tumor-induced modifications to cellular context, or their combination. In this context, tumor-specific promoters, which allow for OV gene expression exclusively in cells exhibiting the activity of cancer-related transcription factors [11,12,13], exemplify the strategy of encoding for selectivity in OV genomes. Conversely, malignancy-induced defects in cell-autonomous immunity, restricting the ability of tumor cells to raise antiviral responses and counter OV infection [7,14,15,16,17,18,19,20,21,22,23,24,25,26], exemplify the contributions of modifications to the cancer-cell context to OV selectivity. Such defects in cell-autonomous immunity commonly arise in the context of the immune editing of tumors, as reductions in the abilities of cancer cells to induce, secrete, or respond to interferon (IFN) may facilitate their evasion from the selective pressures applied by anti-tumor immunity [27,28,29,30,31]. Cancer cells induce defects in IFN signaling and IFN-based antiviral responses through diverse molecular mechanisms. These mechanisms include modifications to the cancer-cell genome (e.g., deletions in the type I IFN gene cluster on chromosome 9), epigenetic silencing of mediators of antiviral responses, and signaling-induced defects to the expression and functions of IFNs and their receptors, pattern-recognition receptors (PRRs) or infection-restricting IFN-stimulated genes (ISGs) [7,14,16,19,20,25,32,33,34,35,36,37,38,39,40,41,42,43]. In addition to the enhancement of the selectivity toward tumor cells, efforts toward making improvements in OV efficacy aim at increasing the abilities of OVs to modify the immune tumor microenvironment and promote anti-tumor immunity. Examples of this latter approach include the arming of OVs with immunostimulatory cytokines and chemokines, including GM-CSF, TNFα, IFNs, and different interleukins (e.g., IL-2, IL-7, or IL-23) [44].

While the therapeutic success of OVs as monotherapies has been limited, they are currently viewed as highly suitable for combination therapies. Prominent in this context are combination regimens of OVs and immune checkpoint inhibitors (ICIs), which aim to harness the immune stimulation via infection and the dis-inhibition of anti-tumor immune responses by ICIs [45]. OV-ICI combinations, involving different OVs and varied tumor types, have been tested in pre-clinical and clinical settings [46,47,48,49,50,51,52]. While pre-clinical studies support the potential of OV-ICI combinations, their clinical benefit has yet to be fully substantiated [51]. An alternative approach for the potentiation of the therapeutic efficacy of OVs involves the combination of OVs and precision-therapy compounds. For example, multiple different oncolytic viruses have been paired with small-molecule kinase inhibitors in pre-clinical settings, yielding promising results for several combinations [53]. While numerous clinical trials involving OVs are being performed, and oncolytic virotherapy received approval in China almost 20 years ago [54], FDA approval has been given only to T-VEC, an oncolytic herpes virus [55].

### 2.2. Microtubule Targeting Agents as Modifiers in the Cancer-Cell Context

Hyper-proliferation is a central component of tumorigenesis [56,57], and microtubule (MT) dynamics are critical for cell division. Over 50 years ago, vinca alkaloids and taxanes were identified as microtubule-targeting agents (MTAs), and since then, the targeting of MTs and their dynamics remains a central component of therapy for a broad range of indications, including hematological malignancies and solid tumors [58,59,60]. In these contexts, MTAs are commonly integrated into combination chemotherapy regimens, e.g., the use of taxanes in adjuvant therapy for numerous types of solid tumors (e.g., breast, ovarian, bladder, or lung cancer) [60]. MTAs exhibit marked structural diversity, and many MTAs that were tested for anti-malignancy therapy potential were isolated from marine and botanical species [60]. An interesting exception is 2-methoxyestradiol (2ME2), a natural metabolite of 17β-estradiol that is generated via the activity of liver enzymes [61,62]. The binding of numerous MTAs to tubulin has been characterized at high resolution by crystallography (reviewed in [63]), resulting in their classification according to their target sites on tubulin (e.g., the colchicine site) and their effect on microtubule polymerization when tested at high concentration (enhancement vs. inhibition). Notably, at lower and therapy-relevant concentrations, both polymerization inhibitors and polymerization enhancers potently suppress microtubule dynamics, resulting in kinetic stabilization of the microtubules without alterations to the overall microtubule polymer mass [64]. As a result, both classes, i.e., MT-polymerization enhancers and MT-polymerization inhibitors, may act similarly to block mitosis, resulting in the arrest of the cell cycle at the spindle assembly checkpoint (SAC) [65]. In addition to exhibiting distinct pharmacokinetic and bioavailability parameters (e.g., the limited bioavailability of 2ME2, [66]), MTAs also differ by (i) susceptibility to clearance via ABC efflux pumps (e.g., the sensitivity of vinca alkaloids [67,68] and taxanes [69,70] to multidrug resistance (MDR) pumps, as opposed to the insensitivity exhibited by other MTAs, such as 2ME2 [71]); (ii) degree of specificity toward tumor cells, relative to non-tumor cells. In this context, the induction of neutropenia [72] limits the tolerability of MTAs. Interestingly, the differential susceptibility of hematopoietic-specific tubulin to MTAs (e.g., insensitivity of hematopoietic β1 tubulin to 2ME2, [73]) may affect the degree by which distinct MTAs induce myelosuppression [73]. MTA-mediated targeting of stromal cells may also yield therapeutic benefits. Examples of this are the MTA-mediated targeting of the tumor vasculature [74,75] or the MTA-mediated enhancement of non-canonical T-cell-mediated cancer-cell killing [76]. The notion of differences in the cellular effects elicited by distinct MTAs is exemplified by the differential attenuation of inflammatory responses in murine macrophages activated with IFN-γ and lipopolysaccharide upon co-treatment with 2ME2, colchicine, or paclitaxel [77]. 2ME2, which excelled in the reduction of inflammation-related phenomena in vitro [77], also exhibited anti-inflammatory activity in animal models of acute lung injury, rheumatoid arthritis, and chronic airway inflammation [77,78,79]. Such differences in immunomodulation may also be of importance in cancer-therapy scenarios.

Perturbation to mitosis progression, in general, and arrest of cancer cells at the SAC, in particular, have profound implications on the activation and function of tumorigenic signaling pathways. The molecular bases of these perturbations include (but are not limited to) alterations to the trafficking and function of signal-transducing receptors [80,81,82,83,84], mitosis-intrinsic modifications to transcription and translation [85,86,87,88,89], and the crosstalk between the mitotic signaling program and other signaling responses. The latter phenomenon is exemplified by CDK1-mediated phosphorylation of hundreds of cellular factors in mitosis [90,91,92], many of which regulate signal transduction pathways. In the context of MTA-mediated cancer therapy, the effects on signaling pathways that mediate cell stress or cell death are of particular interest. Indeed, numerous studies employing different cancer-cell models and distinct MTAs reported on MTA-mediated activation of c-Jun N-terminal kinase (JNK)/stress-activated protein kinase (SAPK) signaling cascade as a critical mechanistic element in the MTA-induced effects, including cell death [93,94,95,96,97,98,99,100,101,102,103,104,105,106,107,108,109,110].

Mitotic failure typically stems from DNA damage, perturbations to spindle formation or dynamics, or defects in the expression or regulation of executors of cell-cycle checkpoints. The outcomes of such failures are heavily influenced by characteristics of the cycling cell, including its ploidy and ability to execute cell-death programs, parameters which are frequently altered in malignant cells. Thus, in cancer therapy, the manners by which tumor cells resolve or tolerate prolonged arrests in mitosis reflect an integration of tumorigenesis-induced defects to checkpoint-mediated regulation of the cell cycle [111], malignancy-induced defects to cell-death pathways [112], and the nature and potency of the insult imposed by the therapeutic agent. The inherent heterogeneity of cancer cells, including the differences among cell lines employed in studies of mitosis-targeting agents, has yielded contrasting results, leading to difficulty in reaching consensual definitions of mitotic catastrophe and the cellular processes it involves [113,114,115,116,117,118]. In this context, and given the unsustainability of maintenance of the mitosis-arrested state over prolonged periods, cells arrested in mitosis may undergo distinct cell-death processes (e.g., autophagy [113], apoptosis [119,120,121,122], necroptosis [123,124], pyroptosis [125], or necrosis [126,127]) or mitotic-slippage [89,128,129,130,131]. The latter phenomenon allows the cancer cell to avoid cell death upon the exit from drug-induced mitotic arrest, contributing in this manner to MTA resistance, chromosomal instability, and aneuploidy. The “choice of fate” between mitotic slippage and cell death is thought to result from a balance between pro-apoptotic caspase activation and cyclin B1 degradation [132,133,134,135]. In the absence of (sufficient) caspase activation, a reduction in cyclin B1 levels below the mitotic exit threshold induces mitotic slippage. Conversely, caspase activation (i.e., breaching of the apoptosis threshold) before (sufficient) cyclin B1 degradation results in mitotic cell death. Of note, JNK is implicated in all of the above-mentioned death-related cellular outcomes which stem from mitotic arrest at the SAC, including autophagy and autophagic cell death [136,137,138,139], apoptosis [140,141,142], necroptosis [143,144], and pyroptosis [145]. In addition to its intimate connection to the cellular responses to stress which may stem from MTA treatments, JNK has also been found to regulate cell-cycle progression [146,147,148], and thus its excessive activation may contribute to MTA-induced mis-regulation of the cell cycle.

A critical and evermore-appreciated component of cancer therapies pertains to their ability to stimulate anti-tumor immunity. MTAs regulate anti-tumor immunity at different levels. Thus, MTAs contribute to anti-tumor immunity through the formation of micronuclei following chromosome missegregation, a phenomenon that differs in efficacy among MTAs and tumor-cell types [149]. A proposed mechanism for the immune stimulation stemming from the formation of micronuclei involves the accumulation of DNA damage within these micronuclei, their rupture, and the induction of inflammatory responses upon exposure of this DNA to cytosolic DNA sensors (cGAS-STING) [150]. Recently, an alternative model was proposed where immune stimulation stemming from docetaxel treatment depended on cGAS-coated chromatin bridges and a cytokinesis-related tension-dependent mechanism [151]. While these scenarios are relevant for cells that escape the SAC, arrest in mitosis per se may modify cell-autonomous immune responses [152]. For example, the DNA sensor cGAS, which is involved in cell-autonomous immune responses to DNA viruses, was found to be inactivated in mitosis due to phosphorylation, binding to chromatin elements, and mitosis-induced vesiculation of the Golgi [153,154,155,156]. Interestingly, in cells arrested in mitosis, low levels of cGAS-induced phosphorylation of IRF3 were proposed to stimulate apoptosis by inhibiting the anti-apoptotic activities of Bcl-xL [157]. In contrast, the double-stranded RNA (dsRNA)-dependent protein kinase (PKR), an additional mediator of cell-autonomous immunity and regulator of inflammation [158], is activated in mitosis via exposure of PKR to endogenous dsRNAs upon nuclear envelope breakdown [159]. Interestingly, PKR was also shown to be phosphorylated by CDK1 and was proposed as a regulator of the response to paclitaxel in ovarian and breast cancer cells via the repression of the expression of the anti-apoptotic protein Bcl2 [160]. A further connection between MTA-mediated arrest in mitosis, IFN, and cell-death pathways is demonstrated by the stimulation of IFN-induced necroptosis, an immunogenic form of cell death, upon a combination of caspase inhibition and MTA-mediated arrest in mitosis [124]. MTAs may also modulate immune responses via direct effects on immune cells, including in manners that may contribute to tumor eradication [76,161].

MTAs may also alter the cellular context of the interphase cells and thus mediate mitosis-independent MTA effects that contribute to the anti-malignancy efficacy of these compounds on one hand, while possibly eliciting side effects on non-dividing healthy cells on the other [109,161,162,163,164,165,166,167]. Mechanistically, perturbations to microtubule dynamics may directly affect intracellular trafficking and thus modify the placement and function of intracellular organelles, with broad implications in the cellular context. Moreover, taxanes were also shown to modulate the intracellular localization of executors of signaling pathways, as exemplified by the inhibition of ligand-induced nuclear translocation of the androgen receptor and of the transcriptional activation of its target genes in prostate-cancer cells [167]. In addition, and in accordance with the roles of microtubules as regulators of cell morphology, processes that involve alterations to this morphology, such as tumor cell invasiveness, were also shown to be affected by MTAs [164].

## 3. Testing of OV–MTA Combinations

**Figure 1:**
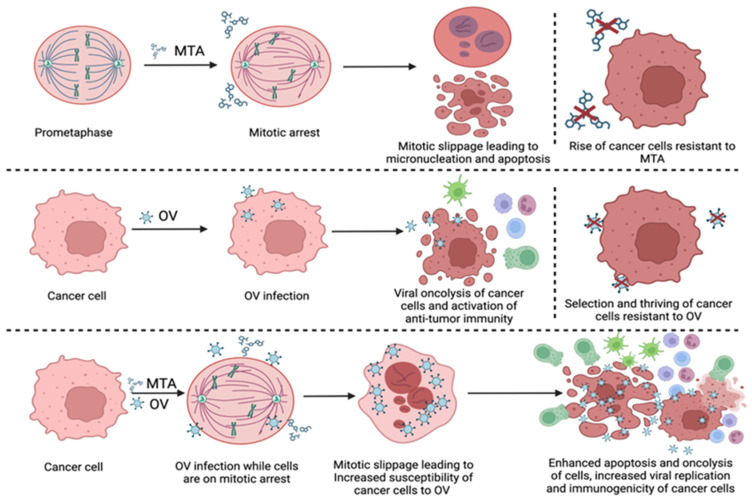
**Mechanisms of action of MTAs, OVs, and their combination for enhancement of tumor clearance.** The top panel schematically depicts events following MTA-based treatment of cancer cells. MTAs arrest cancer cells at the SAC, leading either to apoptosis (mitotic cell death) or to the evasion of cell death by mitotic slippage. The latter phenomenon is associated with acquired resistance to MTAs. The middle panel represents the working principle of OVs, which selectively infect and kill cancer cells and, in doing so, activate anti-tumor immunity. However, cancer cells exhibit differential susceptibility to OVs, and a subset of malignant cells are resistant to this form of therapy. The lower panel schematically shows the potential synergistic effects of MTA-OV combinations. These combinations may lead to increased cell death via the augmentation of different cell death mechanisms, including apoptosis and oncolysis. Moreover, cancer cells treated with MTA-OV combinations may be especially immunogenic due to the combined effects of increased viral replication, its accompanying increase in pathogen-associated molecular patterns, and the enhanced exposure of danger-associated molecular patterns originating from the dying cells. This enhancement in immunogenicity is expected to increase the infiltration of cytotoxic immune cells and promote their anti-tumor activities, leading to improved clearance of malignant cells. **Created with BioRender.com**.

In recent years, several different OVs have been combined with MTAs in a variety of tumor types, in studies ranging from characterizations of infection and cell death of cells in culture to murine pre-clinical models and clinical trials [168,169,170,171,172,173,174,175,176,177,178,179,180,181,182,183,184,185,186,187,188,189,190,191,192,193,194,195]. These studies were not limited to a single type of OV and employed oncolytic variants of adenoviruses [169,170,171,172,173,174,175,176,177,182,183,185,186,192,196,197,198,199,200,201,202,203], reovirus [178,179,180,184,188,191,204], herpes virus [168,181,189,190,205,206,207], vaccinia [193,208], and rhabdoviruses (e.g., oncolytic vesicular stomatitis virus, VSV) [194,195,209]. Several such studies, including the virus, MTA, and cellular models employed, are summarized in Table 1. In a subset of the clinical studies, the OV-MTA combination was supplemented with the nucleoside analog gemcitabine [169,187] or with the inducers of DNA damage, doxorubicin [168,189], or carboplatin [178,188]. These combinations presumably exacerbate cell stress and burden the tumor cell with additional death-promoting stimuli. As such, they may tip the scale of the combinatorial treatment through increases in therapy efficacy and a further reduction in the cancer cell’s ability to develop resistance. Depending on the OV and cancer model, the mechanistic underpinnings associated with OV-MTA therapy efficacy and benefit included the potentiation of viral infection through perturbations to the translation (and thus expression of the protein products) of IFN or antiviral genes [194,195], increased expression of the cellular receptor employed by the virus [177], or the potentiation of cytostatic and cytotoxic phenomena, including mitotic slippage [186,190], apoptosis [168,169,186,191], autophagy [193], and immunogenic cell death (ICD) [192,193]. Of note, ICD and enhanced secretion of cytokines also contribute to non-cell-autonomous benefits of MTA-OV combination through the enhancement of immune stimulation and bystander cytotoxic effects [193,194]. The different scenarios stemming from single vs. combined OV and MTA therapies are schematically depicted in Figure 1.

## 4. Concluding Remarks

The combination of MTAs and OVs brings together two contrasting forms of cancer therapy with the potential to elicit synergistic anti-tumor effects. A subset of MTAs (e.g., taxanes) have been extensively tested and are broadly employed components of the standard of care for many different tumor types. Thus, many of the clinical requirements for using these MTAs are defined. These established conditions of safety and efficacy can also be employed as a basis for combination therapies involving OVs. In contrast to the broad usage and known limitations (e.g., relatively high frequency of toxicity or acquired resistance) of MTAs, the clinical usage of OVs is limited. Thus, many unknowns are still related to the full extent of their potential and/or restrictions as monotherapies or combination therapy agents. Moreover, given the heterogeneity of OVs, the conditions determined for one OV do not apply to a different OV, even for closely related OVs (e.g., distinct clones of the same virus of origin). This poses a considerable challenge, as OVs are highly malleable through reverse genetics, thus stimulating a constant effort of further improvement via the generation of novel clones.

The current state of the oncolytic virotherapy field is characterized by numerous OVs (based on a broad variety of viruses) being under different stages of pre-clinical development, a significantly reduced number of OVs having reached advanced stages of clinical testing, and only a single OV with FDA approval [213]. As detailed above, in vitro and pre-clinical animal studies strongly indicate the potent effects of MTA-OV combinations. Certain phenomena, such as MTA-mediated arrest at the SAC and OV-mediated elicitation of immune-stimulatory antiviral responses, are expected to be common elements of many different OV-MTA combinations. However, viruses (and thus OVs based on these viruses) are very heterogeneous in structure and infection programs, underscoring the potential for specific features of different OV-MTA combinations. For example, viruses exhibit differential dependence on microtubule-based cytoskeletal dynamics or cell-cycle phases and are expected to be differentially affected by MTAs that stabilize or destabilize microtubules. Moreover, MTAs may differ in their ability to ignite distinct sets of signal transduction pathways (e.g., JNK). This too is expected to have differential consequences for the replication of OVs and their abilities to induce oncolysis. As such, fully harnessing the potential of MTA-OV combinations requires optimization of these combinations (what OV and what MTA) to specific tumor contexts. Similarly, given the potential differences in the outcomes of specific MTA-OV combinations, further addition of therapeutic agents (e.g., DNA damaging agents or immune checkpoint inhibitors) may also be optimized with dependence on the MTA and OV employed. Together, the number of different OVs and MTAs generates an extensive number of options that need to be quantitatively assessed to allow for meaningful comparisons and rational decisions regarding the usage of a particular OV-MTA combination. However, laboratories generally study only small selections of OV types, and the comparison of the results between different studies is difficult due to the lack of standardization in the manners of testing of OVs, in general (e.g., how viral preparations are made, or the viral titers which are employed), and OV-MTA combinations, in particular. Thus, coordination between different laboratories in planning and performing such comparative experiments may be required to generate a reliable database of quantitative assessments of OVs and OV-MTA combinations. In this context, collaborative studies aimed at such comparisons should be stimulated and supported financially.

## Figures and Tables

**Table 1:** Studies involving OV-MTA combinations.

Oncolytic Virus	Compound	Cancer Type/Cell Line	Ref.
Oncolytic Measles Virus (OMV)	Vincristine	Human prostate cancer cells (PC3)	[210]
Oncolytic Adenovirus SG600	Vincristine	Human retinoblastoma cells (HXO-RB44)	[196]
Oncolytic adenovirus Ad5	Vincristine	Non-small-cell lung cancer (NSCLC)	[197]
Oncolytic Herpes simplex virus (G207)	Vincristine	Human rhabdomyosarcoma cells (KFR, KF-RMS-1)	[205]
Oncolytic parvovirus H-1PV	Vincristine	Human melanoma cells (Mz-Mel, SK29-Mel-1, SK29-Mel-1.22)	[211]
Oncolytic adenovirus OBP-401	Vinorelbine	Human non-small-cell lung cancer (H1299) and human colorectal carcinoma (SW620) cells	[198]
Oncolytic Vesicular Stomatitis Virus VSV-Δ51	Vinorelbine/colchicine	Human renal carcinoma cells (768-O), a mouse model of triple-negative breast cancer (4T1)	[194]
Oncolytic Herpes Simplex Virus (NV1023) and human IL12 producing variant (NV1042)	Vinblastine	Human prostate cancer cell lines (CWR22, PC3), in vivo xenograft (CWR22)	[181]
Oncolytic Reovirus Type 3 Dearing strain (ReoT3D)	Vinblastine	Non-small-cell lung cancer cells (EKVX, NCL-H322M, NCL-H226)	[204]
Oncolytic Rhabdovirus Maraba MG-1 virus	Paclitaxel	Murine mammary carcinoma cells (4T1, EMT6, EO771), mouse tumor models (4T1, EMT6), human breast cancer cells (S578T, BT-549 and MDA-MB-231)	[209]
Oncolytic adenovirus AD5D24-CpG	Paclitaxel	Human lung cancer cells (A549)	[199]
Oncolytic Herpes simplex virus (G207), Oncolytic Herpes Simplex Virus (NV1023)	Paclitaxel	Human anaplastic thyroid cancer cells (KAT-4, DRO90-1)	[168]
Oncolytic Herpes Simplex virus (G47∆)	Paclitaxel	Human breast cancer (MCF-7 and MDA-MB-468 cell lines)	[206]
Oncolytic Vaccinia virus (vvDD)	Paclitaxel	Human colorectal carcinoma cells (HCT-116), human breast cancer cells (MCF-7), human ovarian cancer cells (UCI-101)	[193]
Oncolytic Vaccinia virus (GLV-1h68)	Paclitaxel	Human pancreatic adenocarcinoma cells (AsPc-1, Panc-1)	[208]
Oncolytic Herpes simplex virus type I (VG161)	Paclitaxel	Mouse breast cancer tumor model EMT6	[207]
Oncolytic adenovirus (dl922-947)	Paclitaxel	Human ovarian cancer cells (A2780CP, IGROV1)	[186]
Oncolytic Herpes simplex virus (G47∆)	Paclitaxel	Human Prostate cancer cells (LNCaP, DU145)	[190]
Oncolytic adenovirus (AdV5/3-D24-ICOSL-CD40L,)	Paclitaxel	Human breast cancer cells (MCF-7, MDA-MB-231, MDA-MB-468)	[200]
Oncolytic adenovirus (OBP-401)	Paclitaxel	Human gastric cancer cells (GCIY, KATOIII)	[183]
Oncolytic adenovirus (Ad-fosARE)	Paclitaxel	Human lung cancer cells (A549, H1299), human cervical carcinoma cells (HeLa, HeLa S3, C-33), human osteosarcoma cells (U-2-OS) human hepatoma cells (HepG2)	[177]
Oncolytic adenovirus (oAd-vp53)	Paclitaxel	Human breast cancer cells (MCF-7, SKBR-3), human ovarian cancer cells (MDAH 2774), human lung cancer cells (H460)	[201]
Oncolytic recombinant measles virus (rMV-BNiP3)	Paclitaxel	Human breast cancer cells (MCF-7, MDA-MB-231)	[212]
Oncolytic adenovirus (PRRA)	Docetaxel	Human prostate cancer cells (CWR22rv, C4-2 cell), CWR22rv subcutaneous tumor xenograft mouse model	[202]
Oncolytic adenovirus (OBP-401)	Docetaxel/vinorelbine	Human non-small cell lung cancer cells (A549, H1299, H226Br), human colorectal carcinoma cells (SW620, DLD-1), human gastric cancer cells (MKN28), human esophageal cancer cells (T.Tn, TE8), human prostate cancer cells (LNCaP) and human hepatic cancer cells (HepG2), human H1299 xenograft mouse model	[198]
Oncolytic adenovirus (ZD55-SATB1)	Docetaxel	Human prostate cancer cells (PC3 and DU145), PC3 xenograft	[203]
Oncolytic Reovirus Type 3 Dearing (ReoT3D)	Docetaxel	Human prostate cancer cells (PC3 and DU145), PC3 xenograft	[191]
